# Publisher's Note: “Enzyme transient state kinetics in crystal and solution from the perspective of a time-resolved crystallographer” [Struct. Dyn. **1**, 024701 (2014)]

**DOI:** 10.1063/1.5016802

**Published:** 2017-11-28

**Authors:** Marius Schmidt, Dilano K. Saldin

**Affiliations:** Physics Department, University of Wisconsin, Milwaukee, Wisconsin 53211, USA

This article was originally published online on 27 March 2014 with several typographic errors in the text. On page 024701-4, paragraph 2, lines 1, 2, 7, 17, and 19; page 024701-5, paragraph 2, line 2; page 024701-7, paragraph 2, line 8, and in Ref. 34, the word “substrate” (“substrates”) should be changed to “substate” (“substates”). AIP Publishing apologizes for this error. The online version of the article was corrected on 16 November 2017.

